# Clinical and Analytical Evaluation of the Alinity m HR HPV Assay within the VALGENT-3 Framework

**DOI:** 10.1128/JCM.00286-21

**Published:** 2021-05-19

**Authors:** Sharonjit K. Dhillon, Anja Oštrbenk Valenčak, Lan Xu, Mario Poljak, Marc Arbyn

**Affiliations:** a Unit of Cancer Epidemiology/Belgian Cancer Centre, Scientific Institute of Public Health, Brussels, Belgium; b Institute of Microbiology and Immunology, Faculty of Medicine, University of Ljubljana, Ljubljana, Slovenia; c School of Public Health, Shanghai Jiao Tong University School of Medicine, Shanghai, China; Cepheid

**Keywords:** Alinity m, VALGENT, HPV genotyping, cervical cancer, human papillomavirus, test validation

## Abstract

Only clinically validated human papillomavirus (HPV) tests should be used in cervical cancer screening. VALGENT provides a framework to validate new HPV tests. In the VALGENT-3 study, the clinical accuracy of the recently launched Abbott Alinity m HR HPV assay (Alinity m) to detect cervical precancerous lesions was assessed against the standard comparator test (Hybrid Capture 2; HC2) and against two previously validated alternative comparator tests (Abbott RealTi*m*e HR HPV and Roche cobas 4800 assays). Validation was conducted using 1,300 consecutive cervical samples from women attending an organized population-based cervical screening program enriched with 300 cytologically abnormal samples. Overall high-risk HPV test concordance was assessed by kappa values; the concordance for HPV-16 and HPV-18 was assessed for Alinity m, RealTi*m*e, and cobas, and the Linear Array (Roche) was used for more detailed genotyping concordance. In the total study population, the relative sensitivity and specificity for cervical intraepithelial neoplasia grade 2 or worse (CIN2+) and CIN3+ of Alinity m compared to HC2 was 1.02 (95% confidence interval [CI], 0.99 to 1.06) and 1.03 (95% CI, 0.99 to 1.06), respectively. The relative specificity for nondiseased subjects (≤CIN1) was 1.01 (95% CI, 1.00 to 1.02) (all *p*_non-inferiority_ ≤ 0.001). Alinity m showed noninferior clinical accuracy among women 30 years or older when cobas or RealTi*m*e was used as a comparator. HPV genotype-specific concordance between Alinity m and the three comparator tests showed excellent agreement, with kappa values ranging from 0.82 to 1.00. In conclusion, Alinity m fulfills the international accuracy requirements for use in cervical cancer screening and shows excellent HPV genotype-specific concordance with three clinically validated HPV tests.

## INTRODUCTION

The strong etiological association recognized between persistent high-risk human papillomavirus (hrHPV) infection and the development of cervical cancer (1, 2) has resulted in an abundance of tests for HPV on the global market (3, 4). Robust evidence from randomized controlled trials has shown that screening based on HPV has greater effectiveness than cervical cytology for decreasing the rate of cervical precancer and invasive carcinoma in primary screening among women 30 years and older (5, 6). As a consequence, a paradigm shift has been occurring in the last decade in many screening programs for cervical cancer away from cytology and toward hrHPV testing (7–13).

More than 220 HPV genotypes have been identified (14). On the basis of association with precursor lesions and cervical cancer, HPV types belonging to the *Alphapapillomaviruses* are grouped into low-risk and high-risk HPV types. The International Agency for Cancer (IARC) considers 12 HPV genotypes (HPV-16, -18, -31, -33, -35, -39, -45, -51, -52, -56, -58, and -59) carcinogenic due to an etiological link with developing cervical cancer and with precursor lesions (group I carcinogens), with HPV types 16 and 18 being the most potent carcinogenic agents, accounting for approximately 70% of all cervical cancers (15–17). Several hrHPV tests target, in addition to 12 hrHPV types, one or two additional HPV types (HPV-66 and HPV-68), although they are considered probably or possibly carcinogenic (IARC group 2A or 2B) (18). Genotyping for at least HPV-16 and HPV-18, and probably some other types, is clinically relevant, allowing for risk-based triage of hrHPV screen-positive women (19, 20). Only a dozen hrHPV DNA tests have been fully clinically validated and meet international consensus requirements for use in primary screening settings (4, 6, 21, 22). The VALidation of HPV GENotyping Tests (VALGENT) is a research framework for comparing and validating HPV tests designed for screening and genotyping according to international validation guidelines (23). This study further evaluates the clinical accuracy of the assay Alinity m HR HPV (Alinity; Abbott Molecular, Des Plaines, IL, USA) (24 and A. Oštrbenk Valenčak, A. Bertram, A. Gröning, M. Poljak, unpublished data), a recently launched hrHPV test with extended genotyping capacity using samples from the third installment in the VALGENT framework (VALGENT-3).

## MATERIALS AND METHODS

### VALGENT-3 panel.

The study population for VALGENT-3 consists of 1,600 samples. Of these, 1,300 consecutive samples were acquired from women 25 to 64 years old who took part in an organized national screening program for cervical cancer in Slovenia (the screening population). Following the VALGENT protocol (23), 300 samples further enriched the study population, collated from women referred to colposcopy after an abnormal cytology result. These 300 samples (the enrichment population) consisted of 100 female patients who had atypical squamous cervical cells with undetermined significance (ASC-US), 100 that had low-grade squamous intraepithelial lesions (LSIL), and 100 that had high-grade squamous intraepithelial lesions (HSIL). Two cervical samples were collected from each patient: a specimen for conventional cytological examination and another specimen that was placed into a ThinPrep PreservCyt solution (Hologic, Marlborough, MA, USA) (25). These second specimens collected in liquid-based cytology medium were transported to the laboratory, anonymously labeled, and divided into multiple aliquots before being stored at −80°C for HPV DNA testing (25).

Ethical approval was obtained for this study from the Slovenian Medical Ethics Committee (consent no. 83/11/09 and 109/08/12).

### HPV tests.

All 1,600 samples were tested with Alinity m, Hybrid Capture 2 HPV DNA test (HC2; Qiagen, Gaithersburg MD), RealTi*m*e High Risk HPV test (RealTi*m*e; Abbott, Wiesbaden, Germany), and cobas 4800 HPV test (cobas; Roche Molecular Systems, Alameda, CA). All tests were performed according to the manufacturer’s instructions.

Alinity m is a fully automated hrHPV test launched in 2019 that targets the conserved L1 region of HPV DNA of 14 hrHPV types. hrHPVs are detected with genotype-specific probes in five distinct channels: HPV-16, HPV-18, HPV-45, group A (HPV-31, -33, -52, and -58), and group B (HPV-35, -39, -51, -56, -59, -66, and -68) (24 and Oštrbenk Valenčak et al., unpublished). The Alinity m test is performed by the Alinity m System, which offers automated continuous random access, and results were obtained from the test software based on comparing the cycle number (CN) values of the specimen for each signal with established signal-specific cutoff values. The human beta-globin gene is used as an internal control for evaluating sample extraction, cell adequacy, and amplification efficiency (24 and Oštrbenk Valenčak et al., unpublished).

HC2 (Qiagen), launched in 1998, is a semiquantitative test and one of two recommended standard comparator tests in the international validation guidelines. HC2 detects 13 genotypes of hrHPV (HPV-16, -18, -31, -33, -35, -39, -45, -51, -52, -56, -58, -59, and 68) was used in this study as a standard comparator test against which the clinical performance of Alinity m was evaluated. Sample testing with HC2 (25) was carried out from December 2009 to September 2010 and from January 2014 to June 2015 on the screening and enrichment population, respectively, within 2 weeks after sample collection.

RealTi*m*e (Abbott), launched in 2008, is an automated multiplex real-time PCR test that targets the L1 region of 14 different hrHPV genotypes and has been clinically validated in several previous studies (25–27). The test allows separate detection of HPV-16 and HPV-18 and aggregate detection of 12 additional hrHPV genotypes, namely, HPV-31, -33, -35, -39, -45, -51, -52, -56, -58, -59, -66, and -68. Testing samples using RealTi*m*e was carried out from December 2009 to September 2010 and from January 2014 to June 2015 on the screening and enrichment population, respectively, within 2 weeks after sample collection.

cobas (Roche), launched in 2011, is a multiplex real-time PCR test that is fully automated and targets the L1 region of 14 different hrHPV genotypes with the same HPV genotyping capability as RealTi*m*e. cobas has been clinically validated in several previous studies (21, 28–31). Testing of the study population with cobas was performed in 2015.

Linear Array (Roche) is a test for HPV that has full genotyping capacity, and it can discriminate between 37 low- and high-risk HPV genotypes that are frequently used for epidemiological and virological studies (32). This study used Linear Array as a comparator test to evaluate genotype-specific concordance between tests beyond HPV-16 and HPV-18. Testing of the study population with Linear Array was conducted in 2016. A previous study (33) clinically validated the Linear Array assay for screening cervical cancer (restricted to 14 hrHPV genotypes).

### Clinical outcomes and statistical analyses.

According to the criteria of the Slovenian program for cervical cancer screening, women are given an immediate colposcopy referral relying on an atypical squamous cell threshold that cannot exclude high-grade lesions or worse (ASC-H) or according to the study protocol if they were positive for HPV-16/18, irrespective of cytology findings. Specimens are taken via punch biopsy during colposcopy from any region suspected of cervical intraepithelial neoplasia (CIN), and certified pathologists with more than 20 years of gynecological pathology experience examine the samples (25).

The diseased group (denominator for clinical sensitivity estimation) included women with histologically confirmed cervical intraepithelial neoplasia grade 2 or worse (CIN2+) and grade 3 or worse (CIN3+). Clinical specificity estimates were calculated for the control or nondiseased group, which included patients who had received two negative cytology results in a row upon enrollment and also at the following screening 12 to 48 months later.

In addition to the standard comparator (HC2), cobas and RealTi*m*e were included as additional comparator tests because they have clinical accuracy similar to that of HC2 and genotyping capacity similar to that of Alinity m. CIN2+ and CIN3+ clinical sensitivity and ≤CIN1 clinical specificity were calculated for Alinity m, HC2, RealTi*m*e, and cobas. We assessed clinical performance for the complete study population, regardless of age, and in patients 30 and older. We used the McNemar (McN) test to compare differences between paired proportions. The noninferior accuracy of Alinity m versus the comparator tests was assessed with the matched noninferior statistic *p*_non-inferiority_ (*p*_ni_) proposed by Tang et al. (34), accepting 0.90 and 0.98 as benchmarks for relative sensitivity.

In addition, kappa (κ) values and McNemar statistics were used to assess genotype-specific concordance for the HPV genotypes common to Alinity m and Linear Array, cobas, and RealTi*m*e (35). The κ value ranges indicated agreement between two assays: 0.0 to 0.20, poor; 0.21 to 0.40, fair; 0.41 to 0.60, moderate; 0.61 to 0.80, good; and 0.81 to 1.0, excellent (36).

The statistical significance level was set at 0.05. Statistical analyses were carried out with STATA version 14 (College Station, TX, USA).

## RESULTS

**FIG 1:**
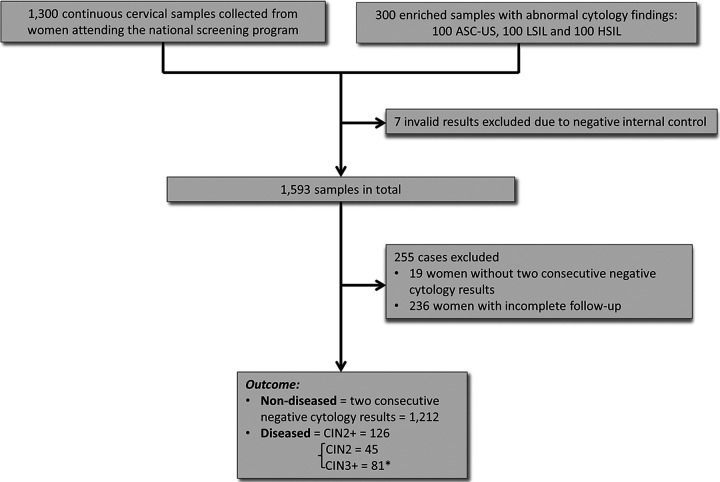
Flowchart explaining selection of nondiseased subjects (≤CIN1) and diseased cases. Women with histologically confirmed CIN2+ and CIN3+ were used as the denominator for sensitivity (*n* = 126) and women with two consecutive negative cytology results (≤CIN1) as the denominator for specificity (*n* = 1,212). *, 81 CIN3+ cases include 1 squamous carcinoma and 1 adenocarcinoma.

Of the 1,600 samples that were tested with Alinity m, seven samples (0.4%) were excluded from further analyses due to negative β-globin results. Figure 1 shows a flow chart presenting the process starting with panel collation of samples and ending with ascertainment of diseased and nondiseased cases. The entire study population comprised 1,593 samples with valid Alinity m results. Of these, 255 women were excluded from the clinical evaluation because they either did not have two consecutive negative cytology results at least 3 years apart (*n* = 19) or had incomplete follow-up (*n* = 236). Thus, 1,212 women with two subsequent negative cytology results within at least 3 years (denominator for the computation of clinical specificity) and 126 patients with CIN2+ (denominator for computation of clinical sensitivity) were used for assessment of clinical accuracy. Of 126 patients with CIN2+, 45 had CIN2 and 81 had CIN3+, as shown in Fig. 1.

### Absolute clinical sensitivity of Alinity m, HC2, cobas, and RealTi*m*e.

Within the total study population, 124/126 CIN2+ and 81/81 CIN3+ cases showed positivity with Alinity m, which corresponds to sensitivity for CIN2+ and CIN3+ of 98.4% (95% CI, 94.4 to 99.8%) and 95.1 (95% CI, 95.5 to 100.0%), respectively (see Table 1). Out of the 1,212 ≤CIN1 results (nondiseased population), 1,104 tested negative with Alinity m, corresponding to a specificity of 91.9% (95% CI, 89.3 to 92.6%). Among women 30 years and older, Alinity m showed a sensitivity for CIN2+ of 99.0% (97/98; 95% CI, 94.4 to 100.0%) and 100.0% (95% CI, 94.6 to 100.0%; 66/66) for CIN3+. Alinity m’s specificity for excluding ≤CIN1 was 93.8% (95% CI, 92.1 to 95.2%; 946/1,009). The accuracy estimates for HC2, cobas, and RealTi*m*e are shown in Table 1 for the entire study population as well as for women 30 years or older.

**TABLE 1:** Sensitivity for CIN2+ and CIN3+ and specificity for ≤CIN1 of Alinity m, HC2, RealTi*m*e, and cobas in the total study population and in women ≥30 years old

Test and group	Sensitivity for CIN2+			Sensitivity for CIN3+			Specificity for ≤CIN1		
	%	95% CI	*n*/*N*	%	95% CI	*n*/*N*	%	95% CI	*n*/*N*
Total study population
Alinity m	98.4	94.4–99.8	124/126	100.0	95.5–100.0	81/81	91.1	89.3–92.6	1,104/1,212
HC2	96.0	91.0–98.7	121/126	97.5	91.4–99.7	79/81	90.1	88.3–91.7	1,092/1,212
RealTi*m*e	96.8	92.1–99.1	122/126	98.8	93.3–99.9	80/81	91.7	90.1–93.2	1,112/1,212
cobas	96.0	91.0–98.7	121/126	97.5	91.5–99.7	79/81	91.4	89.7–93.0	1,100/1,203
Women ≥30 yr old
Alinity m	99.0	94.4–100.0	97/98	100.0	94.6–100.0	66/66	93.8	92.1–95.2	946/1,009
HC2	95.9	89.8–98.9	94/98	97.0	89.5–99.6	64/66	92.9	91.1–94.4	937/1,009
RealTi*m*e	96.9	94.4–100.0	95/98	98.5	94.6–100.0	65/66	94.5	92.7–95.7	954/1,009
cobas	96.9	91.3–99.4	95/98	97.0	89.5–99.6	64/66	94.1	92.5–95.5	943/1,002

### Relative sensitivity and specificity of Alinity m compared to the standard comparator (HC2).

In the entire study population, Alinity m had a somewhat higher sensitivity than HC2: 1.02 (95% CI, 0.99 to 1.06) for CIN2+ and 1.03 (95% CI, 0.99 to 1.06) for CIN3+. The specificity for ≤CIN1 of Alinity m was marginally higher than that of HC2 (1.01 [95% CI, 1.00 to 1.02]). The sensitivity for CIN2+ and CIN3+ and specificity for ≤CIN1 of Alinity m was noninferior to that of HC2 (*p*_ni_ = 0.0001, *p*_ni_ = 0.0006, and *p*_ni_ < 0.0001), respectively, for the entire study population. Stratifying the analyses for women 30 years or older yielded similar results (see Tables S1 and S2 in the supplemental material).

### Relative sensitivity and specificity of Alinity m versus RealTi*m*e and cobas.

The relative sensitivity for CIN2+ of Alinity m versus RealTi*m*e and cobas in the entire study population was 1.02 (95% CI, 0.99 to 1.04) and 1.02 (95% CI, 1.00 to 1.05), respectively. The relative specificity for ≤CIN1 of Alinity m was 0.99 (95% CI, 0.98 to 1.00) compared to RealTi*m*e and 1.00 (95% CI, 0.99 to 1.01) compared with cobas (Tables S1 and S2).

### Test genotyping concordance between Alinity m versus cobas, RealTi*m*e, and Linear Array assays.

Tables 2 to 4 show the overall hrHPV and HPV genotype-specific concordance between Alinity m and the three comparator assays. Excellent concordance was observed for the overall detection of the 14 hrHPV genotypes as well as for the detection of HPV-16, HPV-18, and HPV-16/18 for Alinity m versus cobas, Alinity m versus RealTi*m*e, and Alinity m versus Linear Array, whereby HPV-16, HPV-18, and HPV-16/18 results include both single or multiple infections. The concordance for HPV-45 between Alinity m and Linear Array was 99.7% (κ = 0.81). When genotype detection by Alinity m versus cobas and Alinity m versus RealTi*m*e was stratified by clinical setting (screening and enriched populations), excellent agreement was observed again (kappa range, 0.82 to 1.00) (HPV-16, HPV-18, and HPV-16/18) and, overall, 14 hrHPV genotypes (Table S3).

**TABLE 2:** Overall hrHPV and type-specific concordance between Alinity m and cobas assessed on all samples with valid HPV results included in the VALGENT-3 panel (*n* = 1,584)

HPV type^*b*^	Value (no.) by test result						Concordance (%)	Kappa^*a*^
	Alinity m^+^	cobas^+^	Alinity m^+^/cobas^+^	Alinity m^+^/cobas^−^	Alinity m^−^/cobas^+^	Alinity m^−^/cobas^−^		
14 hr types	349	345	328	21	17	1,218	97.60	0.9299
HPV-16	116	114	112	4	2	1,466	99.62	0.9719
HPV-18	34	35	32	2	3	1,547	99.68	0.9259
HPV-16/18	145	145	139	6	6	1,449	99.31	0.9581

a Kappa legend (adapted from Landis and Koch [36]) for levels of agreement: 1.00 ≥ κ > 0.80, excellent; 0.80 ≥ κ > 0.60, good; 0.60 ≥ κ > 0.40, moderate; 0.40 ≥ κ > 0.20, fair; 0.20 ≥ κ > 0.00, poor.

b HPV-16, -18, and -16/18 indicate that particular HPV types are present as a single or multiple infections.

**TABLE 3:** Overall hrHPV and type-specific concordance between Alinity m and RealTi*m*e assessed on all samples with valid HPV results included in the VALGENT-3 panel (*n* = 1,593)

HPV type^*b*^	Value (no.) by test result						Concordance (%)	Kappa^*a*^
	Alinity m^+^	RealTi*m*e^+^	Alinity m^+^/RealTi*m*e^+^	Alinity m^+^/RealTi*m*e^−^	Alinity m^−^/RealTi*m*e^+^	Alinity m^−^/RealTi*m*e^−^		
14 hr types	349	324	324	25	8	1,236	97.93	0.9384
HPV-16	116	113	113	3	0	1,477	99.81	0.9859
HPV-18	34	31	30	4	1	1,558	99.69	0.9215
HPV-16/18	145	139	138	7	1	1,454	99.56	0.9729

a Kappa legend (adapted from Landis and Koch [36]) for levels of agreement: 1.00 ≥ κ > 0.80, excellent; 0.80 ≥ κ > 0.60, good; 0.60 ≥ κ > 0.40, moderate; 0.40 ≥ κ > 0.20, fair; 0.20 ≥ κ > 0.00, poor.

b HPV-16, -18, and -16/18 indicate that particular HPV types are present as a single or multiple infections.

**TABLE 4:** Overall hrHPV and type-specific concordance between Alinity m and Linear Array assessed on all samples with valid HPV results included in the VALGENT-3 panel (*n* = 1,593)

HPV type^*b*^	Value (no.) by test result						Concordance (%)	Kappa^*a*^
	Alinity m^+^	LA^+^	Alinity m^+^/LA^+^	Alinity m^+^/LA^−^	Alinity m^−^/LA^+^	Alinity m^−^/LA^−^		
14 hr types	349	332	332	17	7	1,237	98.49	0.9555
HPV-16	116	113	112	4	1	1,476	99.81	0.9859
HPV-18	34	34	31	3	2	1,556	99.62	0.9098
HPV-16/18	145	141	138	7	3	1,452	99.44	0.9653
HPV-45	13	14	11	2	3	1,577	99.69	0.8132
HPV group A	138	137	128	10	9	1,446	98.81	0.9244
HPV group B	122	122	113	9	9	1,462	98.87	0.9201

a Kappa legend (adapted from Landis and Koch [36]) for levels of agreement: 1.00 ≥ κ > 0.80, excellent; 0.80 ≥ κ > 0.60, good; 0.60 ≥ κ > 0.40, moderate; 0.40 ≥ κ > 0.20, fair; 0.20 ≥ κ > 0.00, poor.

b HPV-16, -18, and -16/18 indicate that particular HPV types are present as a single or multiple infections. HPV group A includes HPV-31, -33, -52, and -58; HPV group B includes HPV-35, -39, -51, -56, -59, -66, and -68.

## DISCUSSION

Because international and national evidence-based recommendations continue to propose replacing cytology with HPV testing as a primary tool for screening for cervical cancer, validating novel HPV tests based on international criteria using established comparative frameworks (such as VALGENT) is deemed vital for global high-quality cervical cancer screening efforts and the cervical cancer 2030 elimination goals proposed by the World Health Organization (37).

In addition to detecting 14 hrHPV genotypes, the Alinity m assay provides distinct information on the three most carcinogenic HPV genotypes (HPV-16, -18, and -45) and separates the four subsequently most carcinogenic genotypes into group A (HPV-31, -33, -52, and -58), covered by the nonavalent HPV vaccine, from seven other less carcinogenic HPV genotypes (HPV-35, -39, -51, -56, -59, -66, and -68). This study showed Alinity m’s clinical sensitivity and specificity for detecting CIN2+ in patients 30 years or older to be 99.0% (95% CI, 94.4 to 100.0%) and 100.0% (95% CI, 94.6 to 100.0%), respectively. Compared with the standard comparator test, HC2, in this study, Alinity m demonstrated noninferior CIN2+ sensitivity and specificity in both women 30 years and older as well as in the entire study population. The results of this study confirm those generated in the other Alinity m validation study, in which the clinical accuracy of Alinity m was assessed on 3,145 women 30 years or older and clinical sensitivity for CIN2+ and specificity for ≤CIN1 were 100.0% and 92.4%, respectively (24). Therefore, the results of this study additionally confirm that Alinity m meets international criteria for accuracy as a primary screening test for cervical cancer.

In addition to the standard comparator assay (HC2), this study provides comparison data for Alinity m’s performance against three other clinically validated HPV assays: RealTi*m*e, cobas, and Linear Array. Compared to these three clinically validated HPV assays, noninferior accuracy of Alinity m for CIN2+ and CIN3+ was observed. A previous validation study showed noninferior clinical sensitivity and specificity of Alinity m compared to HC2 and performance of Alinity m comparable to that of HC2, cobas, and RealTi*m*e in terms of 3-year negative predictive value (e.g., 3-year cumulative incidence of CIN2+ lesions after the initial negative screening result) (24). In particular, women who showed baseline hrHPV negativity had a smaller risk of CIN2+ at 3 years without regard for the hrHPV assay that was used (Alinity m [0.04], HC2 [0.08], cobas [0.04], or RealTi*m*e [0.04]) compared to those that had a normal baseline cytology (0.65) (24). In addition, baseline positivity for HPV-16/18 infection (regardless of the hrHPV assay used) correlated with significantly higher 3-year risk for CIN2+ or CIN3+ (24). Another study, performed on 4,334 women attending population-based cervical cancer screening, showed that Alinity m has a noninferior clinical sensitivity and specificity compared to cobas in primary screening settings (Oštrbenk Valenčak et al., unpublished).

In addition to clinical accuracy, Alinity m displays excellent intertest genotyping concordance overall and for the identification of individual HPV genotypes compared to cobas, RealTi*m*e, and Linear Array. The research findings that have accumulated in the past years, coupled with recent U.S. FDA approval of the BD Onclarity HPV (Becton, Dickinson and Company, BD Life Sciences–Integrated Diagnostic Solutions, Sparks, MD) assay for extended genotyping, suggest that extended genotyping will play a significant role in clinical practice in the future, in particular for populations with high vaccine coverage for HPV. Because Alinity m showed excellent performance in every clinical and analytical evaluation published to date and, in addition to aggregate information for 14 hrHPV genotypes, provides separate information for all hrHPV genotypes covered by the bivalent, quadrivalent, and nonavalent HPV vaccines, it can be deemed an important tool in possible new management algorithms for hrHPV risk-based screening for primary cervical cancer. Unfortunately, performance of hrHPV assays is the main focus of the current international validation guidelines (22), and although they are urgently needed, no defined validation criteria for HPV genotyping assays as well as guidelines for use of partial and/or extended HPV genotyping as a triage tool are available and widely accepted in the HPV scientific community. In addition to the announced update of international validation guidelines for evaluation of hrHPV assays (22 and M. Arbyn, M. Simon, E. Peeters, L. Xu, C. J. Meijer, J. Berkhof, unpublished data), the U.S. FDA recently issued an executive summary on how to move forward and improve the evaluation of hrHPV assays (38). According to this U.S. FDA document, the new approaches in evaluation of hrHPV assays should take into account the following: broader knowledge of cervical carcinogenesis, decreased incidence and prevalence of HPV vaccine-targeted hrHPV infections due to HPV vaccination, evolving screening and patient management guidelines, and clinical study design, particularly the benefits and risk of enrichment studies using specimens collected from referral populations.

Although in this study we evaluated concordance for HPV-16, HPV-18, and HPV-16/18 among different assays, the proper validation judgment of genotype-specific performance was not possible from our data, which is one of the main limitations of our study. Thus, it would be interesting to analyze pooled genotyping data from all VALGENT iterations (VALGENT 1 to 4) in a separate study to assess different triage options in HPV-based primary cancer screening settings, including partial and extended genotyping. Thus far, only a single pooled analysis nested in the VALGENT framework on the accuracy of HPV-16/18 genotyping to triage women with LSIL was performed. It clearly showed that the presence of HPV-16/18 justifies immediate referral to colposcopy, and that women with LSIL carrying other HPV types cannot be returned to routine screening and require further active monitoring (39, 40). With pooling of data from the present study and other published studies from all four VALGENT iterations, we predict that sufficient statistical power will be achieved to perform further detailed assessment of partial and extended HPV genotyping for triage in primary cervical cancer screening settings. Another potential limitation of our study is the use of bio-banked specimens, where the quality of specimens may deteriorate over time, which could generate a disadvantage to HPV assays that are evaluated years after the collection of the specimens. However, within the VALGENT framework, especially in VALGENT-3, thus far, we did not observe any lower accuracy of HPV assays in correlation to duration of sample archiving (see Table S4 in the supplemental material). Namely, several hrHPV DNA assays validated in VALGENT-3 showed excellent cross-sectional performance, although individual head-to-head evaluations were performed in different laboratories and at different time points after sample collection. VALGENT-3 showed, for the first time, that if appropriately aliquoted and stored, cervical samples collected in ThinPrep PreservCyt solution can be used for clinical validation of hrHPV DNA assays for at least 10 years after collection (and most probably much longer), opening the possibility of the production of high-quality and long-lasting quality assurance panels.

In conclusion, on the basis of the results of this evaluation study and previous ones (24 and Oštrbenk Valenčak et al., unpublished), Alinity m meets all of the requirements for use in primary cervical cancer screening.
