# Supplementary material for: Clinical and Analytical Evaluation of the Alinity m HR HPV Assay within the VALGENT-3 Framework
Source: J Clin Microbiol. 2021 May 19;59(6):e00286-21. doi: 10.1128/JCM.00286-21 (PMC8316144; doi:10.1128/JCM.00286-21)
Supplement: Supplementary file 1 [file jcm.00286-21_suppl1.pdf]

TABLE S1 Relative sensitivity and specificity of Alinity m compared to (i) HC2, (ii) RealTime, and (iii) cobas in the total study population and in women 30 years or older.

**Alinity m vs. HC2**	**Outcome**	**Relative sensitivity**	**Relative specificity**	p_{McN}[a]	p_{Ni}[b]
Total study population	CIN2+	1.02 (0.99–1.06)		0.3750	< 0.0001
	CIN3+	1.03 (0.99–1.06)		0.5000	0.0006
	≤ CIN1		1.01 (1.00–1.02)	0.0578	< 0.0001
Women ≥ 30 years	CIN2+	1.03 (0.99–1.08)		0.3750	0.001
	CIN3+	1.03 (0.99–1.08)		0.5000	0.001
	≤ CIN1		1.01 (1.00–1.02)	0.0555	< 0.0001
**Alinity m vs. RealTi*me***					
Total study population	CIN2+	1.02 (0.99–1.04)		0.5000	< 0.0001
	CIN3+	1.01(0.99–1.04)		1.000	0.001
	≤ CIN1		0.99 (0.98–1.00)	0.0736	0.005
Women ≥ 30 years	CIN2+	1.02 (0.99–1.05)		0.5000	0.001
	CIN3+	1.02 (0.99–1.05)		1.000	0.0019
	≤ CIN1		0.99 (0.98–1.00)	0.0325	0.0123
**Alinity m vs. cobas**					
Total study population	CIN2+	1.02 (1.00–1.05)		0.2500	< 0.0001
	CIN3+	1.03 (0.99–1.06)		0.5000	0.0004
	≤ CIN1		1.00 (0.99–1.01)	0.3359	0.0024
Women ≥ 30 years	CIN2+	1.02 (0.99–1.05)		0.5000	0.0004
	CIN3+	1.02 (0.99–1.05)		1.000	0.0019
	≤ CIN1		1.00 (0.99–1.01)	0.4913	0.0019

[a] p_{McN} = p value for McNemar's test; [b] p_{Ni} = p value for the non-inferiority test

TABLE S2 Paired test results of Alinity m high risk HPV for a) diseased cases (CIN2+) and b) non- diseased (≤ CIN1) cases in total study population with HC2, RealTi*me* and cobas4800.

Diseased cases (CIN2+) in total study population				
**Index Test**	**Reference Test**		**Total**	**P_{Ni}[a]**
	HC2 +	HC2 -		
Alinity m+	120	4	124	
Alinity m -	1	1	2	
	121	5	126	< 0.0001
	RealTi*me*+	RealTi*me*-		
Alinity m+	122	2	124	
Alinity m -	0	2	2	
	122	4	126	< 0.0001
	cobas 4800+	cobas 4800-		
Alinity m+	121	3	124	
Alinity m -	0	2	2	
	121	5	126	< 0.0001

Non-diseased cases (≤ CIN1) in total study population				
**Index Test**	**Reference Test**		**Total**	**P_{Ni}[a]**
	HC2 +	HC2 -		
Alinity m +	94	14	108	
Alinity m -	26	1078	1104	
	120	1092	1212	< 0.0001
	RealTi*me*+	RealTi*me*-		
Alinity m+	94	14	108	
Alinity m -	6	1098	1104	
	100	1112	1212	0.005
	cobas 4800+	cobas 4800-		
Alinity m +	92	16	108	
Alinity m -	11	1084	1095	
	103	1100	1203	0.0024

[a] p_{Ni} = p value for the non-inferiority test

TABLE S3 Overall hrHPV and type-specific concordance between a) Alinity and RealTi*me*; b) Alinity and cobas stratified by the screening and enriched population.

**a) Alinity vs. RealTi*me***								
	**Total study population**				**Screening**		**Enrichment**	
	**All ages**		**Women ≥ 30 years**		**Women ≥ 30 years**		**Women ≥ 30 years**	
**HPV types**	Agreement	Kappa	Agreement	Kappa	Agreement	Kappa	Agreement	Kappa
14 hr types	97.9%	0.938	98.2%	0.939	98.5%	0.902	96.3%	0.917
HPV-16	99.8%	0.986	99.9%	0.987	99.8%	0.946	100.0%	1.000
HPV-18	99.7%	0.922	99.6%	0.876	99.7%	0.822	99.3%	0.949
HPV-16/18	99.6%	0.973	99.5%	0.967	99.5%	0.907	99.7%	0.993
**b) Alinity vs. cobas**								
	**Total study population**				**Screening**		**Enrichment**	
	**All ages**		**Women ≥ 30 years**		**Women ≥ 30 years**		**Women ≥ 30 years**	
**HPV types**	Agreement	Kappa	Agreement	Kappa	Agreement	Kappa	Agreement	Kappa
14 hr types	97.6%	0.930	97.8%	0.927	98.0%	0.875	97.3%	0.938
HPV-16	99.6%	0.972	99.9%	0.987	99.1%	0.946	100.0%	1.000
HPV-18	99.7%	0.926	99.8%	0.929	99.8%	0.888	99.0%	0.928
HPV-16/18	99.3%	0.958	99.6%	0.973	99.6%	0.927	99.0%	0.978

TABLE S4 Timeline of collection and testing of samples within VALGENT-3.

	screening population (n = 1,300)	enrichment population (n = 300)
Sample collection period (mm/yyyy)	12/2009 – 9/2010	1/2014 – 6/2015
Testing period (mm/yyyy)		
Hybrid Capture 2	12/2009 – 9/2010	1/2014 – 6/2015
RealTi*m*e High Risk HPV	12/2009 – 9/2010	1/2014 – 6/2015
cobas 4800 HPV	12/2015 –7/2016	
Alinity m HR HPV	8/2018 – 9/2018	
